# Engineering Supramolecular Hydrogen Bonding Interactions into Dynamic Covalent Polymers To Obtain Double Dynamic Biomaterials

**DOI:** 10.1021/jacs.4c15102

**Published:** 2025-05-23

**Authors:** Jasper G. M. Aarts, Maritza M. Rovers, Martin G. T. A. Rutten, Patricia Y. W. Dankers

**Affiliations:** 1 Institute for Complex Molecular Systems (ICMS), Eindhoven University of Technology, Eindhoven 5600 MB, The Netherlands; 2 Department of Biomedical Engineering, Eindhoven University of Technology, Eindhoven 5600 MB, The Netherlands; 3 Department of Chemical Engineering & Chemistry, Eindhoven University of Technology, Eindhoven 5600 MB, The Netherlands

## Abstract

Inspired by dynamic systems in nature, we can introduce dynamics into synthetic biomaterials through dynamic covalent bonds or supramolecular interactions. Combining both types of dynamic interactions may allow for advanced and innovative networks with multiple levels of dynamicity. Here we present two types of solid materials consisting of either dynamic covalent imine bonds or a combination of these dynamic covalent bonds with supramolecular hydrogen bonding ureido-pyrimidinone (UPy) units to obtain double dynamic materials. We showed the facile synthesis and formulation of both materials at room temperature. The thermal and physical properties of each material are highly tunable by altering the ratio and type of cross-linker. Interestingly, we showed that minimal amounts of UPy units result in a drastic increase in material mechanics. Furthermore, we show that both types of materials are suitable as biomaterials through functionalization with cell-adhesive peptides, through either a dynamic covalent imine bond or a supramolecular UPy moiety.

## Graphical abstract

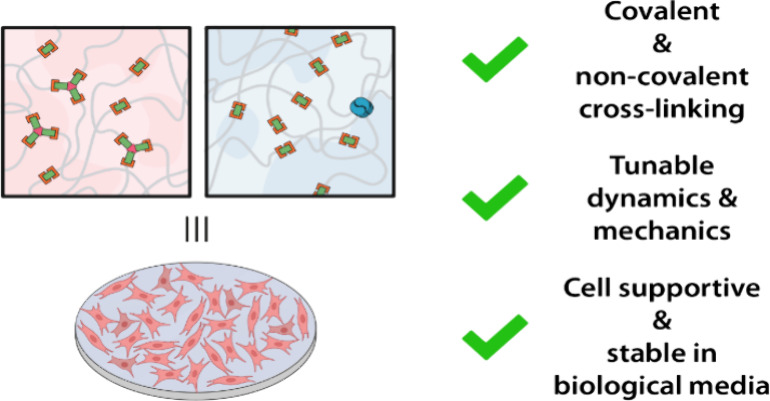

## Introduction

Inspired by the natural extracellular matrix (ECM), dynamic biomaterials have been shown to be beneficial for cell culture.^1,2^ During the development of novel biomaterials, several design criteria must be taken into account. For instance, the physical and chemical properties of the ECM should be mimicked to support cells, but biochemical properties should also be similar to the ECM to promote the adsorption of proteins and other biomolecules. Therefore, polymeric materials may be combined modularly with certain additives to give that material the desired properties, like bioactivity,^3–5^ mechanical strength,^6^ antifouling,^7,8^ or antimicrobial properties,^9–12^ depending on its application.

Different types of chemistry may be used to achieve such suitable biomaterials that mimic the dynamics and mechanics of the natural ECM. One type of bond that can be employed is the dynamic covalent bond. These covalent bonds can be dynamically exchanged via a chemical reaction.^13,14^ The bonds are stable under static conditions, though external stimuli like pH, solvent, light, and temperature can cause bond exchange.^15,16^ When these types of bonds are used to create a polymer network, a covalent adaptable network (CAN) is formed that can change its topology through molecular rearrangements. This response is attained without causing irreversible harm to the network structure because it preserves the initial bond density and enables the material to rearrange.^17–20^ Some great examples of CANs were shown, both in bulk and as ECM mimicking hydrogels.^21–34^ Leibler et al. presented a breakthrough in 2011 with a new type of covalent cross-linked network that formed the bridge between thermoplastics and thermosets, vitrimers. This polyester network was mechanically stable and fully solvent resistant but also able to flow upon heating with the incorporation of a suitable catalyst.^21^ The beauty of vitrimers is that not only ester bonds are capable of this type of behavior but also different types of bonds, as was later shown by for instance Bowman and co-workers. One example is their thiol-thioester exchange mechanism, where they showed that this exchange was possible in the presence of a base or nucleophile, resulting in much faster stress-relaxation when compared to the control material.^29^ Anseth et al. showed that covalent adaptable networks comprised of water-soluble poly­(ethylene glycol) or hyaluronic acid functionalized with dithiolane groups were able to form hydrogels suitable for cell culture, yet maintaining the same dynamic nature as CANs in bulk.^34^

Supramolecular assemblies possess similar features as dynamic covalent bonds; however these arise from noncovalent interactions like hydrogen bonds, van der Waals forces, host–guest assemblies and π–π interactions, or combinations of those.^35–37^ Examples of supramolecular motifs are cyclodextrins,^38^ peptide amphiphiles (PA),^39^ bis-ureas,^40^ and ureido-pyrimidinones (UPy).^41^ The mechanical properties of low molecular weight polymers functionalized with supramolecular motifs can be improved significantly by these reversible and dynamic interactions.^42,43^ In addition, additives can easily be incorporated in these materials, using the same supramolecular moiety as the bulk material.^44–47^ Several researchers have shown the potential of supramolecular chemistry as a tool for the formulation of fibrous assemblies for biomedical applications.^39,48–53^ Meijer et al. thoroughly investigated the supramolecular benzene-1,3,5-tricarboxamide (BTA) moiety over the past few years. It forms supramolecular fibers through π-π stacking, and is stabilized by triple hydrogen bonding.^51^ Stupp et al. showed the potential of PAs as scaffolds for bone marrow derived stem and progenitor cells. These PAs form nanofibers in water through hydrophobic interactions. In combination with the bioactive cyclic RGDfK (Arg-Gly-Asp-phe-Lys; cRGD) peptide sequence,^54–57^ it was shown that the nanofibers enhanced viability, proliferation, and adhesion of these cell types.^47^ Alternatively, we showed the potential of the supramolecular UPy motif, which can dimerize via quadruple hydrogen bonding, while π–π interactions induce lateral stacking of the dimers which is further stabilized by flanking hydrogen bonded urea groups.^41,58,59^ These UPy fibers can form hydrogels through cross-linking with bifunctional UPy molecules and act as a synthetic ECM.^6,57,60^

Combining dynamic covalent and supramolecular chemistry to create double dynamic polymers may be remunerative, as these multiple levels of dynamicity give responses to different environmental cues and can be tuned accordingly. One of the earlier examples of such double dynamic polymers, or double dynamers, was shown by Lehn et al. in 2005.^61^ Here, a linear double dynamic polymer, consisting of both dynamic covalent acylhydrazones and supramolecular hydrogen bonded cyanuric acid wedges, was synthesized and analyzed in solution. They later investigated several double dynamic systems, both in solution and in bulk.^62–64^ Odriozola and co-workers showed the potential of these systems through the synthesis of an elastomer based on a double dynamic material consisting of both disulfide bonds and urea groups.^65^ Feringa et al. showed with the use of a single molecule of acylhydrazine-based 1,2-dithiolane the formation of a dual dynamic network of dynamic covalent disulfide bonds and supramolecular H-bonds.^66^ In a similar approach, Otto and co-workers used a benzoic dithiol and a hydrazide functional group to formulate fibers in solution.^67^ Heuts and Sijbesma and co-workers formulated a covalent supramolecular dual motif within one functional group. The 1,2,3,4-benzene tetramide unit acts as a dynamic covalent cross-linker, as well as a supramolecular stacking motif.^68^

Here, we combine two dynamic interactions into one polymer network, which can be modularly functionalized using both types of interactions: dynamic covalent and supramolecular. A linear prepolymer was synthesized through the imine bond formation of a poly­(tetrahydrofuran) (pTHF) terminated diamine and bifunctional salicylaldehyde derivative. These were modularly cross-linked using either the dynamic covalent trifunctional tris­(2-aminoethyl)­amine (TREN) or the supramolecular amine functionalized UPy (UPy-amine) (Figure 1). The thermal properties, mechanical behavior, and the dynamicity using stress-relaxation rheological measurements were assessed for both types of materials. Finally, bioactive cRGD was incorporated through either dynamic covalent or supramolecular (UPy-cRGD) interactions, respectively, to assess the materials’ biocompatibility.

**Figure 1:**
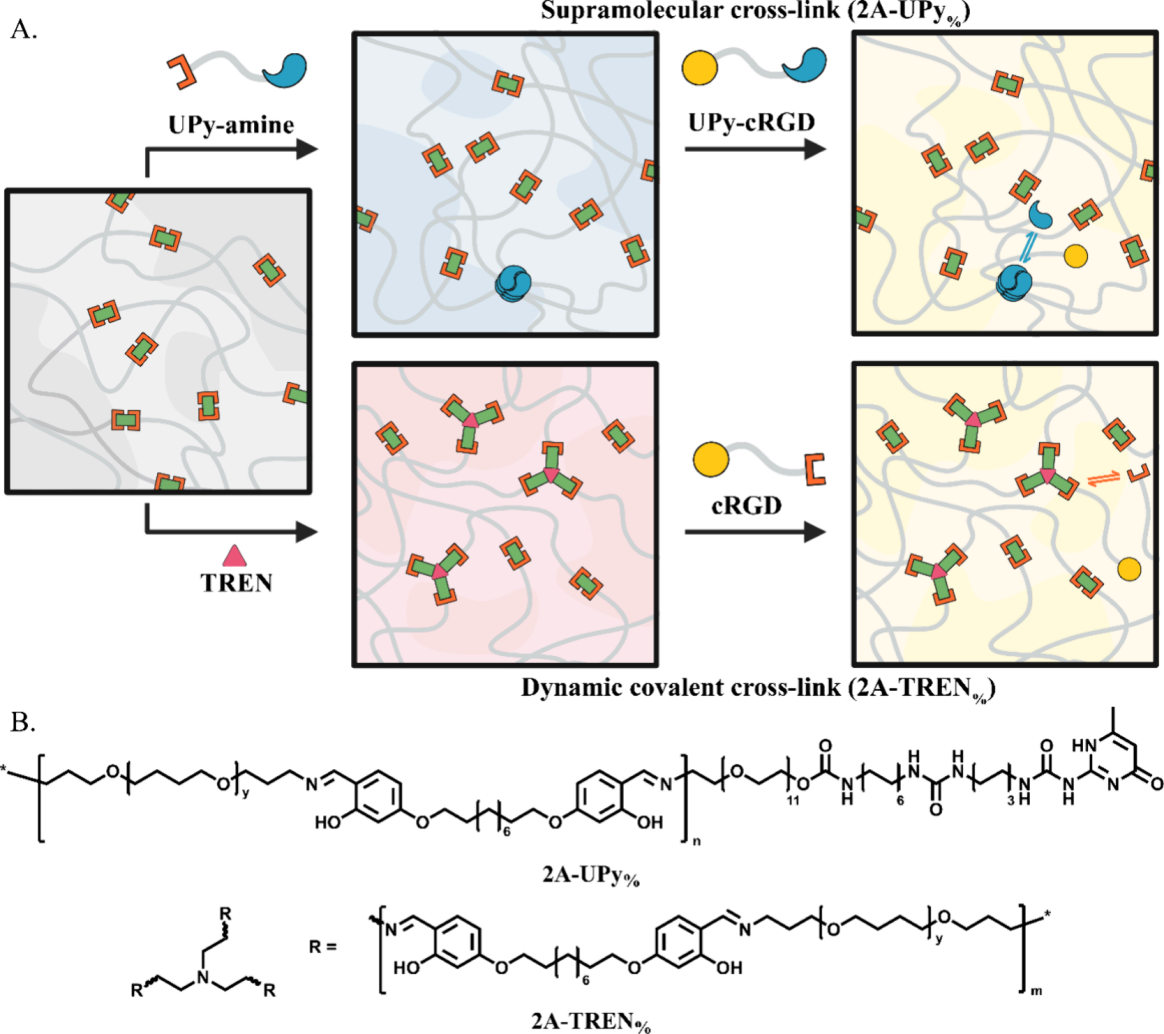
(A) Schematic illustration of the formulation of supramolecular (UPy) and dynamic covalent (TREN) cross-linked networks. (B) Chemical structures of hybrid supramolecular (**2A-UPy**_**%**_) and dynamic covalent (**2A-TREN**_**%**_) networks used in this work.

Our approach allows for the tweaking of material properties, which can cross-link using two different methods. The comparison of these different types of cross-linking allows us to alter the mechanics and dynamics within polymer networks as well as investigate their importance at the cell–material interface.

## Results and Discussion

The synthesis of dialdehydes **1** and **2** was performed using Williamson etherification in acetone for 24 h (Figure 2A). ^1^H NMR, ^13^C NMR, and HMBC NMR as well as MALDI-TOF spectrometry confirmed the pure product formation (Figures S1 and S2).

**Figure 2:**
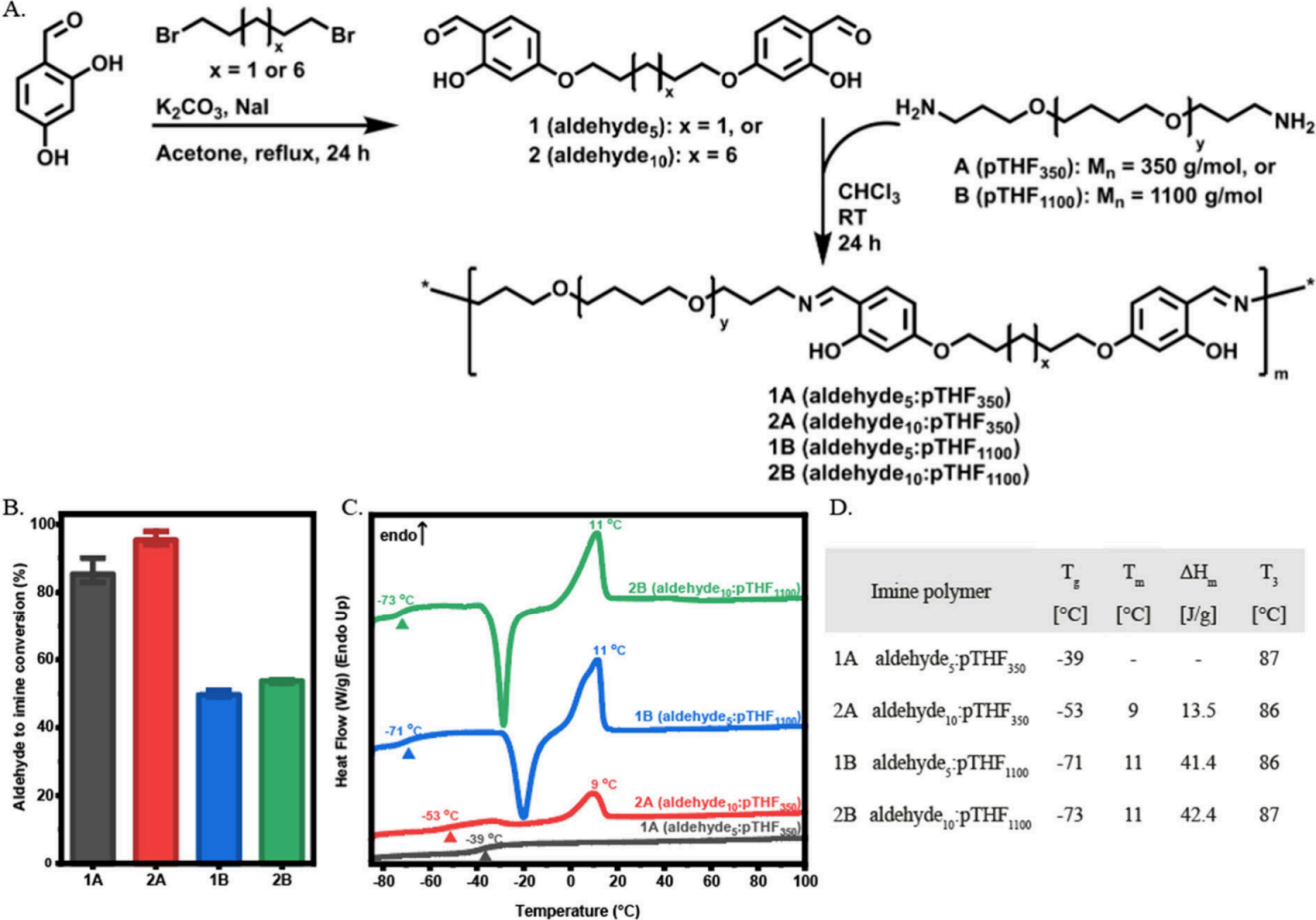
Synthesis, conversion, and thermal properties of polymers **1A**, **2A**, **1B**, and **2B**. (A) Synthetic scheme of **1**, **2**, **1A**, **2A**, **1B**, and **2B**. (B) Conversion of **1A**, **2A**, **1B**, and **2B** after 24 h, as determined by **^1^** H NMR in CDCl_3_. (C) Traces (2nd heating run, 10 °C/min) and (D) thermal properties, determined by DSC.

Next, diamine **A** or **B** was reacted with dialdehyde **1** or **2** in equimolar amounts to form four linear imine polymers (Figure 2A). To confirm the imine formation, the reaction was followed using ^1^H NMR (Figure 2B) with the imine signal appearing while the signal of the aldehyde and amine decreased after 24 h. For polymers **1A** and **2A**, this resulted in conversions of 85% and 95%, respectively. For polymers **1B** and **2B**, a conversion of respectively 50% and 54% was observed. This may be attributed to the longer pTHF chains, which are more coiled, and therefore, the reactive amine group is less available. The imine polymers were annealed at 100 °C and analyzed by Raman spectroscopy where the vibration bands characteristic of the aldehyde stretch at 1651 cm^–1^ disappeared and the imine vibration band at 1629 cm^–1^ appeared, further indicating the successful formation of the linear imine polymers **1A**, **2A**, **1B**, and **2B** (Figure S3).

The thermal behavior of the imine polymers was then assessed using differential scanning calorimetry (DSC) at a rate of 10 °C/min. (Figure 2C). **1A** shows a glass transition (*T*_g_) at −39 °C and no melting temperature (*T*_m_), and thus is an amorphous polymer. **2A** shows a *T*_g_ of −53 °C which is higher than the *T*_g_’s of polymers **1B** and **2B** at −71 and −73 °C, respectively. Furthermore, **1B**, **2A** and **2B** are semicrystalline polymers, as can be seen from the recrystallization and *T*_m_ corresponding to a crystalline domain. For **1B** and **2B** the enthalpy change of the *T*_m_ is significantly larger than that of **2A** (Figure 2D), which is a result of the longer pTHF chain in these polymers.^69^

Interestingly, all four polymers show a small endotherm between 86 and 88 °C. The polymers were subjected to another heating run at 40 °C/min (Figure S4) and here the signal became more evident. In addition, a rheological temperature sweep was performed on **2A** from 30 to 120 °C (Figure S5) and here the storage modulus shows a slight increase between 40 and 80 °C from 66 to 68 kPa, respectively. However, from 80 to 120 °C a steeper increase was observed to a maximum measured storage modulus of 78 kPa at 120 °C which indicates further annealing of the polymer.

**2A** and the trifunctional amine TREN were reacted to form dynamic covalent polymer networks, where molar equivalents of 5%, 20%, and 33% of TREN with respect to **2A** were chosen to create a set of materials with increasing cross-linking density (Scheme 1, Table 1). All reactions were followed using ^1^H NMR, showing the disappearing signals of the amines and aldehydes and the appearance of the imine signal. DOSY NMR confirmed the incorporation of TREN into the polymer network by the decreased diffusion coefficient of the signals corresponding to TREN (Figure 3A).

**Scheme 1:**
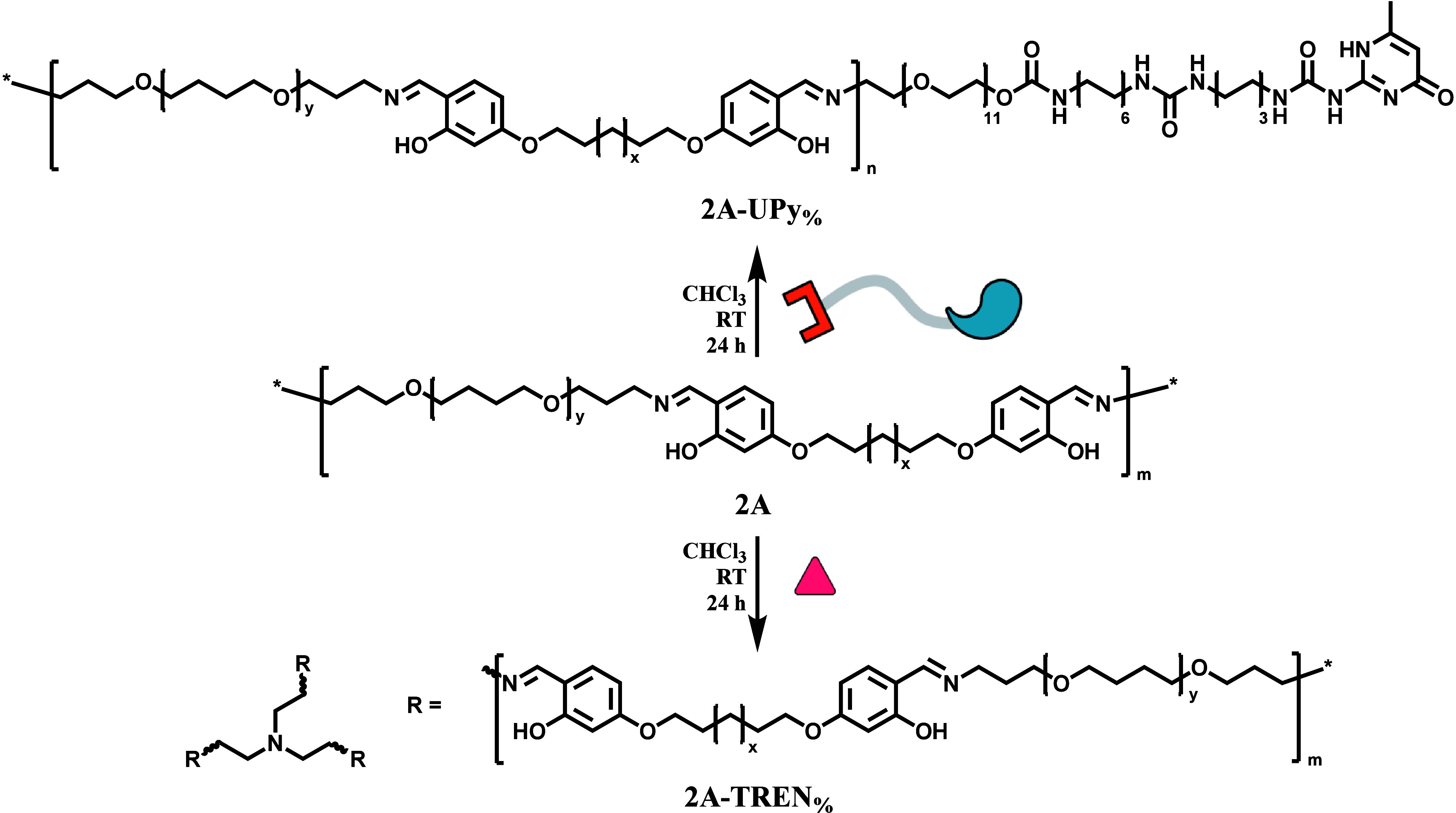
Synthetic Scheme of Supramolecular Cross-Linked **2A-UPy**_**%**_ and Dynamic Covalent Cross-Linked **2A-TREN**_**%**_

**Table 1:** Formulation of Linear (**2A**), Dynamic Covalent (**2A-TREN_%_**), and Supramolecular (**2A-UPy_%_**) Biomaterials

	**2** (aldehyde_10_)		**A** (pTHF_350_)		TREN		UPy-amine	
	mM	equiv of CHO	mM	equiv of NH_2_	mM	equiv of NH_2_	mM	equiv of NH_2_
**2A**	71.0	2.00	71.0	2.00				
**2A-TREN_5_**	71.0	2.00	65.7	1.85	5.3	0.15		
**2A-TREN_20_**	71.0	2.00	49.7	1.40	21.3	0.60		
**2A-TREN_33_**	71.0	2.00	35.5	1.00	35.5	1.00		
**2A-UPy_5_**	71.0	2.00	69.2	1.95			1.8	0.05
**2A-UPy_10_**	71.0	2.00	67.5	1.90			3.6	0.10
**2A-UPy_20_**	71.0	2.00	63.9	1.80			7.1	0.20
**2A-UPy_40_**	71.0	2.00	56.8	1.60			14.2	0.40

**Figure 3:**
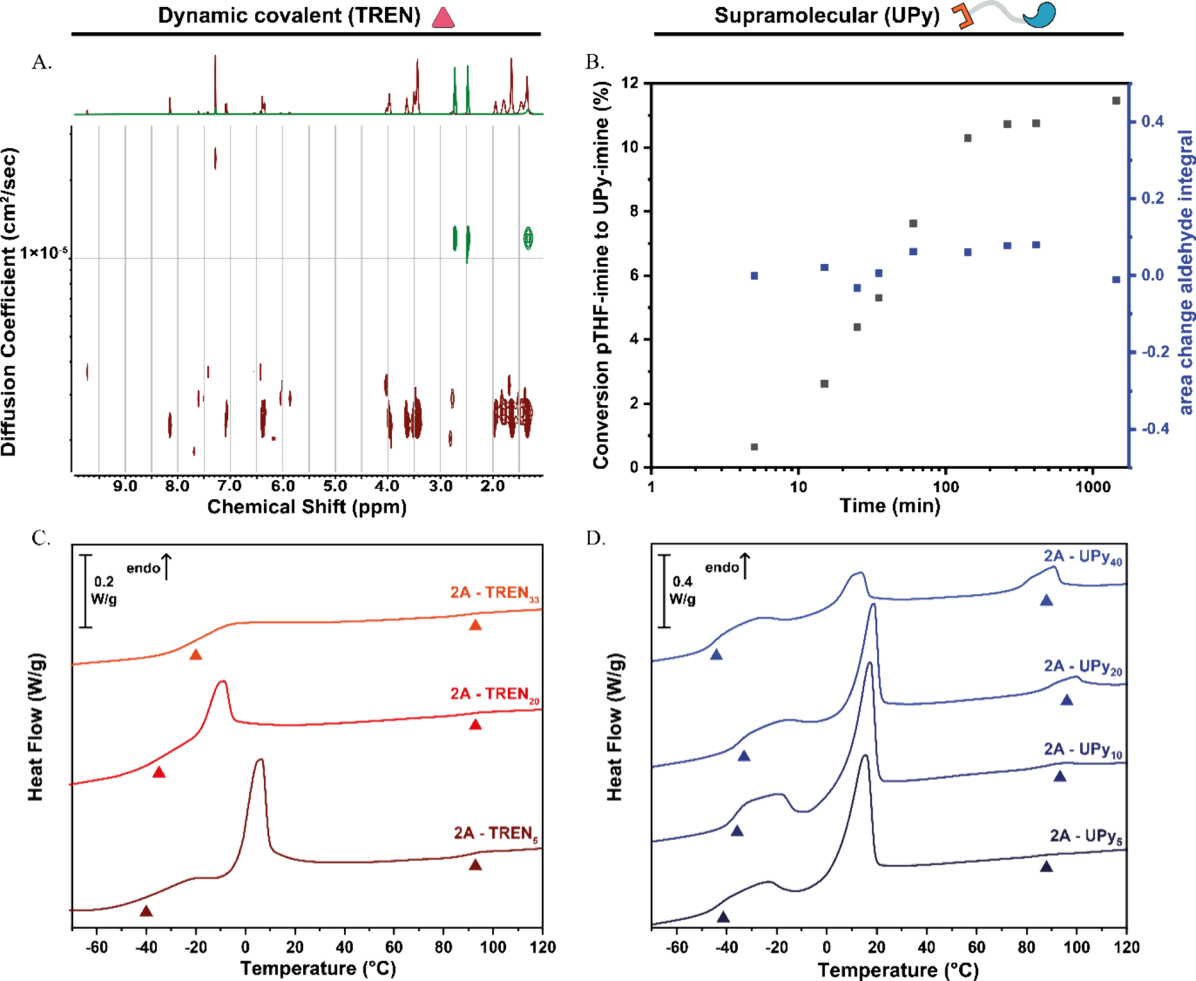
(A) DOSY NMR of pure TREN (green) and **2A-TREN**_**20**_ (red) in CDCl_3_. (B) Conversion of the pTHF-imine to the UPy-imine bond determined by **^1^** H NMR. DSC traces (2nd heating run) of (C) **2A-TREN**_**5**_ until **2A-TREN**_**33**_ at 40 °C/min and (D) **2A-UPy**_**5**_ until **2A-UPy**_**40**_ at 10 °C/min.

DSC at a scanning rate of 40 °C/min revealed the *T*_g_ of the cross-linked polymers (Figure 3C and Table 2). As expected, an increase in the *T*_g_ was observed with a higher cross-linking density,^70^ from −53 °C for linear polymer **2A** to −21 °C for **2A-TREN**_**33**_. The DSC graphs showed a decrease in *T*_m_ when the cross-linking density is increased as well as a decrease in enthalpy change. This is probably due to the lower weight percentage of **A** in the higher cross-linked networks. In addition, the same endotherm was observed for all cross-linked polymers between 92 and 94 °C.

**Table 2:** Thermal Properties of Polymers **2A-TREN_%_** and **2A-UPy_%_** ^a^

	*T*_g,THF_	Δ*C*_p,THF_	*T*_m,THF_	Δ*H*_m,THF_	*T*_3_	Δ*C*_p_	*T*_m,UPy_	Δ*H*_m,UPy_
	[°C]	[J/g·°C]	[°C]	[J/g]	[°C]	[J/g·°C]	[°C]	[J/g]
**2A-TREN_5_**	–29	0.40	7	15.4	94	0.07		
**2A-TREN_20_**	–30	0.28	–9	5.8	93	0.05		
**2A-TREN_33_**	–21	0.43			92	0.04		
**2A-UPy_5_**	–42	0.36	15	17.2			89	0.2
**2A-UPy_10_**	–36	0.33	17	16.4			94	0.8
**2A-UPy_20_**	–35	0.27	18	11.5			99	3.4
**2A-UPy_40_**	–45	0.33	13	3.4			91	4.2

a Second heating run; **2A-TREN_%_**, 40 °C/min; **2A-UPy_%_**, 10 °C/min.

After synthesis of **2A**, the supramolecular UPy-amine was added to the reaction mixture (Scheme 1), ranging from 5 to 40 mol % (Table 1). This way, supramolecular cross-links were introduced into the material through hydrogen bonding rather than through covalent bonds. The imine–amine exchange reaction between **2A** and UPy-amine was followed with ^1^H NMR over time (Figure S6). A new signal attributed to imine bound UPy appeared at 8.16 ppm and increased over time. In addition, the area of the residual aldehyde signal (9.70 ppm) did not change. This showed that the exchange reaction was taking place and after about 100 min the conversion reached a plateau, showing an equilibrium (Figure 3B).

Introducing UPy-amine into polymer **2A** is proposed to have an effect on the behavior of the material by creating a double dynamic covalent/supramolecular network. As such, the thermal behavior was analyzed using DSC (Figure 3D and Table 2). With the addition of UPy-amine, the polymer’s *T*_g_ increased from −53 °C without UPy-amine to between −45 and −35 °C with UPy-amine which is an indication of a higher cross-linking density, caused by the dimerization and stacking of the UPy moieties. Also, a decrease in enthalpy change was observed for the melting transition assigned to the crystalline melt of the pTHF chain. This was expected, since by design, an increase of UPy-amine correlates to a decrease in pTHF chains, resulting in a lower amount of THF crystals in the material. Furthermore, an additional melting peak was observed in **2A-UPy**_**5**_ until **2A-UPy**_**40**_ between 89 and 99 °C with an increased enthalpy change at higher UPy-amine concentration, which was ascribed to the crystalline melt of the UPy moieties. To verify this, atomic force microscopy (AFM) was performed on **2A-UPy**_**20**_ (Figure S7). Here, **2A-UPy**_**20**_ shows the presence of phase separated domains and thus that such crystalline domains are indeed present in the material.

Following the synthesis and annealing at 100 °C, **2A**, **2A-TREN**_**20**_, and **2A-UPy**_**20**_ were processed into discs by compression molding for rheological studies at temperatures ranging from 30 to 80 °C. Polymers **2A**, **2A-TREN**_**20**_, and **2A-UPy**_**20**_ all show viscoelastic behavior, exhibiting both elastic and viscous properties that show a strong dependency upon frequency. Especially *G*″, which decays at lower frequency and is more dominant at higher frequencies (Figure S8).

The storage (*G*′) and loss (*G″*) moduli of **2A**, **2A-TREN**_**20**_, and **2A-UPy**_**20**_ are shown in Figure 4A,B. A slight decrease in *G*′ is observed for polymer **2A** with an increase in temperature, from ∼45 kPa at 30 °C to ∼39 kPa at 80 °C, however not significant. The loss modulus decreased much faster when the temperature was increased, from ∼8.6 to ∼1.1 kPa, respectively. As a result, the value of tan­(δ) (Figure 4C) is higher at low temperatures (0.33 at 30 °C), showing tunable viscoelastic behavior, whereby the material becomes more elastic at elevated temperatures (∼0.10 at 80 °C).

**Figure 4:**
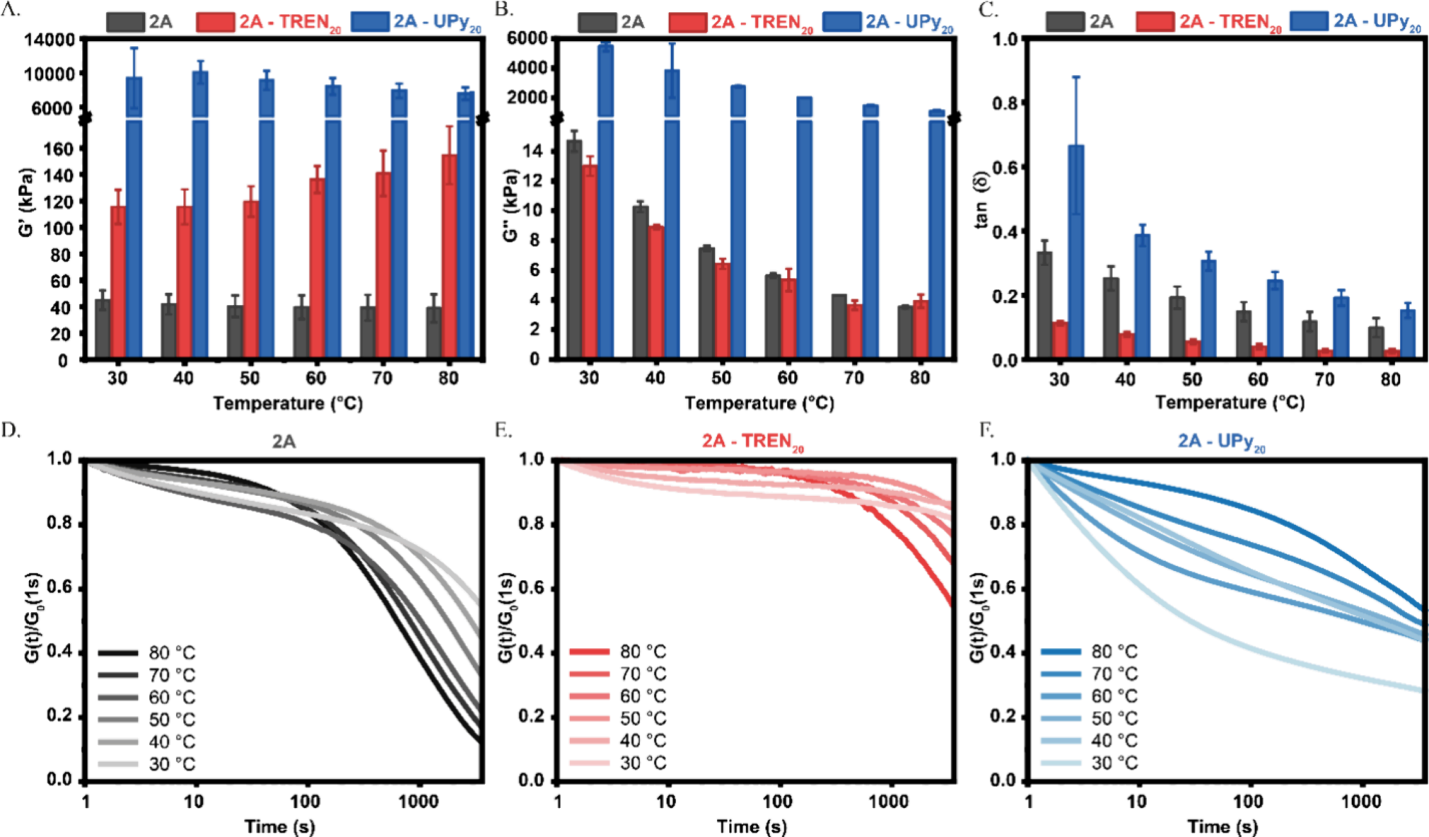
Rheological characterization of **2A** (gray), **2A-TREN**_**20**_ (red), and **2A-UPy**_**20**_ (blue). Measurements were performed between 30 and 80 °C, with (A) storage modulus (*G*′), (B) loss modulus (*G*″), and (C) the loss factor tan­(δ) showing the ratio of viscous to elastic response, obtained at 1 rad/s and 1% strain. Stress–relaxation plot of (D) **2A**, (E) **2A-TREN_20_**, and (F) **2A-UPy_20_**, measured at 1% strain.

The storage modulus of the dynamic cross-linked network **2A-TREN**_**20**_ increases with an increase in temperature, from ∼116 kPa at 30 °C to ∼155 kPa at 80 °C and is higher than that of **2A**, which is a clear indication that the linear polymer was cross-linked by TREN. The loss modulus, just as with **2A**, decreased between 30 and 80 °C from ∼13 kPa to ∼3.9 kPa. This also resulted in a decreasing tan­(δ) as the temperature increased, from ∼0.17 at 30 °C to almost fully elastic at ∼0.03 at 80 °C.

Changing the cross-link chemistry also influences the bulk mechanics of the material. Both *G*′ and *G*″of **2A-UPy**_**20**_ are higher than for **2A-TREN**_**20**_. The values for *G*′ range between 9.4 and 7.6 MPa from 30 to 80 °C, an 80-fold increase at 30 °C and a 50-fold increase at 80 °C. On the other hand, tan­(δ) is higher for **2A-UPy**_**20**_ than **2A-TREN**_**20**_. **2A-UPy**_**20**_ has a tan­(δ) of 0.66 at 30 °C and 0.15 at 80 °C, which also shows a decrease at elevated temperatures but does not become as highly elastic as **2A-TREN**_**20**_. We propose that this huge difference is due to the reinforcements made by crystalline aggregates of the UPy moiety that are capable of cross-linking more than just three polymer chains, which is the case for TREN.

Next, the dynamic behavior of polymers **2A**, **2A-TREN**_**20**_, and **2A-UPy**_**20**_ was studied with stress relaxation experiments (Figure 4D–F). 1% strain was applied, and the stress decay was monitored over a period of 1 h.

**2A** and **2A-TREN**_**20**_ show faster stress relaxation at longer time scales (>100s) upon an increase in temperature, as expected for dynamic covalent materials. Also, the initial modulus of **2A-TREN**_**20**_ at 80 °C (155 kPa) is shown to be more than 3 times higher than that of **2A** at 30 °C (45 kPa), which is consistent with the expectation that the cross-linked network is harder to deform. Furthermore, the normalized relaxation modulus of **2A** after 1 h is significantly lower than that of **2A-TREN**_**20**_ at all temperatures measured, which is explained by the lack of cross-linkers present in the linear **2A**. Both **2A** and **2A-TREN**_**20**_ show a bimodal relaxation modulus. The first relaxation is observed in the initial 100 s, which is most prominent at lower temperatures and decreases at higher temperatures. The second relaxation is found after 100 s and is inversely related to the temperature, with more relaxation at higher temperatures and less relaxation at lower temperatures. These distinct relaxation moduli indicate that there are additional relaxation mechanisms present in addition to the dynamic imine exchange happening within these materials. We hypothesize that the absence of the first relaxation at higher temperatures originates from noncovalent π–π stacking that dissociates at elevated temperatures and therefore does not participate in the relaxation at these temperatures.

**2A-UPy**_**20**_ shows similar changes in stress relaxation processes upon elevated temperature yet more abrupt. At 30 °C relaxation occurs primarily in the first 100 s (60% relaxation), in contrast between 40 and 70 °C, only 26% to 40% of total stresses had been relaxed after 100 s. With a temperature of 80 °C, only 15% of stresses had been relaxed in the first 100 s, whereas more stress is relaxed after 100 s. Thus, the attribution of relaxation at longer time scales (>100 s) becomes more pronounced with an increase in temperature. At lower temperatures these long time scales are less pronounced (13% relaxation after 100 s at 30 °C) while they play an important role at higher temperatures (31% relaxation after 100 s at 80 °C). We propose that the aggregates formed by the UPy moieties contribute to the first relaxation at lower temperature significantly, but at higher temperatures the UPy crystals dissociate and the contribution becomes less. The second relaxation process is a result of the imine exchange of the linear polymer in **2A-UPy**_**20**_.

After solvent casting and annealing of **2A-TREN**_**20**_ and **2A-UPy**_**20**_, NHDFs (10 000 cells/cm^2^) were seeded onto the four materials, cultured for 24 h, and stained for the nucleus (cyan), f-actin (red), and focal adhesions (green). **2A-TREN**_**20**_ and **2A-UPy**_**20**_ are both cell compatible (Figure 5A,B), though slight differences at the cell-material interface were observed. **2A-TREN**_**20**_ shows elongated cells with clear focal adhesions at the cell–material interface, indicating that the cells are strongly attached to the material. **2A-UPy**_**20**_, however, shows less elongated cells that are more densely packed. The cells grow on top of each other rather than in a monolayer, and the cytoskeleton staining is less fiber-like and more homogeneously distributed. These results indicate that even though cells are present, there is less affinity for the cells to interact with **2A-UPy**_**20**_ materials than with **2A-TREN**_**20**_, and there are more cell–cell interactions in the former.

**Figure 5:**
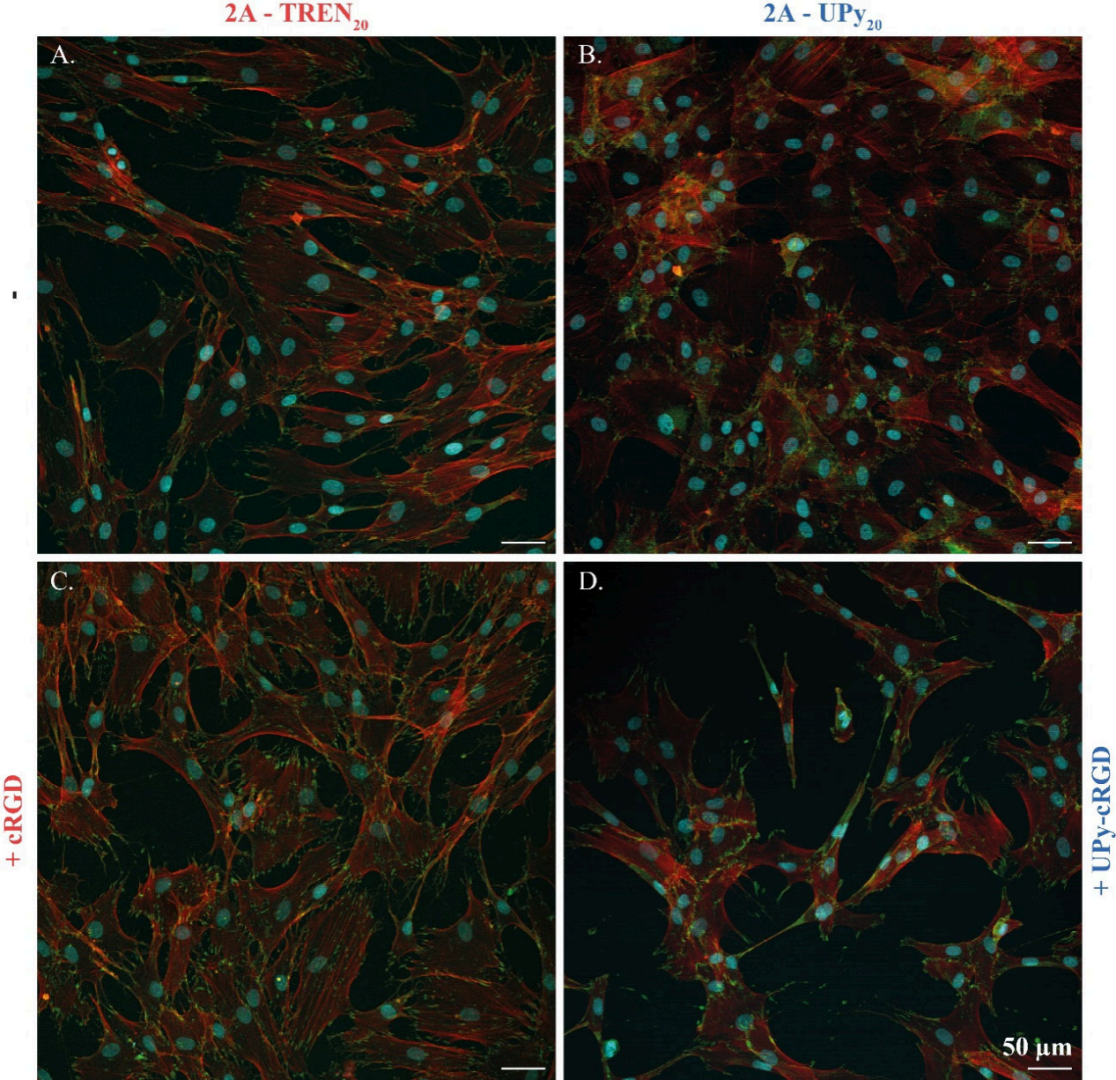
HNDFs cultured for 24 h on (A) **2A-TREN**_**20**_, (B) **2A-UPy**_**20**_, (C) **2A-TREN**_**20**_ + 5 mol % cRGD, and (D) **2A-UPy**_**20**_ + 5 mol % UPy-cRGD, with f-actin (red), nucleus (cyan), and focal adhesions (green). Scale bars: 50 μm.

Therefore, to follow the adhesion of single cells without cell–cell contact, the cells were seeded onto the materials at a lower seeding density (3000 cells/cm^2^) and fixated at time points of 5, 8, and 24 h (Figure S9). These results show that cells grown on **2A-UPy**_**20**_ form clusters, remain round, and do not spread on their own. Cells cultured on **2A-TREN**_**20**_ are distributed over the materials and show an elongated morphology, which can already be seen after 8 h of culturing, all in alignment with above-mentioned results at a higher seeding density.

The dynamically cross-linked **2A-TREN**_**20**_ and supramolecular cross-linked **2A-UPy**_**20**_ were modified by the addition of 5 mol % cRGD or UPy-cRGD, calculated from the repeating unit of **2A**, respectively. As such, bioactivity was incorporated into both networks but with a different type of chemistry and dynamicity. cRGD was incorporated through the dynamic exchange of the amine on the lysine with the imine bonds in the cross-linked network, whereas UPy-cRGD was incorporated through hydrogen bonding with the UPy moieties present in **2A-UPy**_**20**_. By introducing these bioactive components, changes were observed (Figure 5C,D). For **2A-TREN**_**20**_ with cRGD, cells spread out more and had more focal adhesions compared to those of pristine **2A-TREN**_**20**_. With the addition of UPy-cRGD to **2A-UPy**_**20**_ a similar trend was observed, where cells on the material with UPy-cRGD were more elongated, yet there were less cells.

## Conclusions

Two fully synthetic, dynamic biomaterial systems were synthesized starting from one linear dynamic imine base polymer. The base polymer was then cross-linked using the same dynamic covalent imine bonds or using supramolecular UPy interactions, creating a double dynamic network. Depending on the type and amount of cross-linking chemistry, we demonstrated the ability to increase and modulate the glass- and melting transition through the introduction of minimal amounts of cross-linker, with increasing effects at increasing cross-linker concentration. Moreover, we showed that the type of cross-linking strongly affects the mechanical properties (storage and loss modulus) and dynamic behavior (stress relaxation) of these materials.

Compared to the linear polymer, the dynamic cross-linked materials showed an increased glass transition and storage modulus as well as slower stress relaxation. The UPy cross-linked material on the other hand shows an additional crystalline UPy domain, a 50-fold increase in storage modulus, and faster stress relaxation compared to the dynamic cross-linked **2A-TREN_20_**. Finally, we demonstrated that the bioactive cRGD ligand can be incorporated into these materials by using either dynamic covalent imine bonds or supramolecular UPy interactions. All four materials synthesized, with or without cRGD, were cell compatible. Overall this study showed that the cross-linker type drastically changes the mechanical properties of a material, yet no significant difference is observed in the cell–material interactions.

## Experimental Section

### Materials

All reagents and chemicals were obtained from commercial sources at the highest purity available and used without further purification, unless stated otherwise. All solvents were of analytical reagent (AR) quality and were purchased from Biosolve. Automated column chromatography was performed using Biotage SNAP-KP SIL cartridges.

### Instrumentation

#### NMR Spectrosopy

NMR spectra were recorded on a Bruker 400 MHz Ultrashield spectrometer (400 MHz for ^1^H NMR; 100 MHz for ^13^C NMR). The deuterated solvents used are indicated in each case. Chemical shifts (δ) are expressed in parts per million and are referred to the residual peak of the solvent peak. Multiplicity is abbreviated as s, singlet; d, doublet; t, triplet; dd, doublet of doublets; and m, multiplet.

#### Matrix Assisted Laser Desorption/Ionization (MALDI)

MALDI mass spectra were obtained on a Bruker autoflex speed spectrometer using α-cyano-4-hydroxycinnamic acid and 2-[(2*E*)-3-(4-*tert*-butylphenyl)-2-methylprop-2-enylidene]­malononitrile (DCTB) as matrices.

#### Raman Spectroscopy

Raman spectroscopy and analysis was performed using a WITec alpha300 R Raman microscope equipped with a charge-coupled device (CCD) camera (WITec GmbH), a 785 nm laser, a 10× Zeiss EC Epiplan-Neofluar DIC, and a spectrograph with a 600 g mm^–1^ grating for spectral acquisition. An acquisition time of 2 s per spectrum and a laser power of 50 mW were used. Spectral preprocessing and analysis were performed using Project FIVE 5.3 Plus software (WITec GmbH). The data were inspected for cosmic rays, background shape-corrected, and normalized.

#### DSC

DSC data were collected on a DSC Q2000 instrument from TA Instruments and calibrated with an indium standard. The samples (5–10 mg) were weighed directly into an aluminum pan and hermetically sealed. The samples were initially heated to 150 °C and then subjected to two cooling/heating cycles from −85 to 150 °C with a rate of 10–40 °C min^–1^. The data that are presented represent the second heating cycle.

#### Rheology

Rheological measurements were carried out on a TA Instruments Dynamic Hybrid Rheometer 3 equipped with an 8 mm flat stainless steel plate–plate geometry. For measurements, the sample (disk; height = 1 mm, diameter = 8 mm) was transferred to the Peltier plate at 20 °C and the geometry was slowly lowered until it made full contact with the sample (verified via an increase in the axial force). Measurements were started at 30 °C by measuring the complex modulus G* (γ = 0.01, ω = 1 rad/s) until a stable plateau modulus was obtained to ensure no effects of loading on the sample (typical time of 30 min). Subsequent frequency sweep measurements were performed at ω = 0.1 rad s^–1^ to 100 rad s^–1^, at a strain of γ = 0.01. Afterward stress-relaxation was measured by applying a strain of γ = 0.01 with a strain rise time of 0.09 s. The stress was monitored and was normalized using the stress generated after 1 s as starting point. Thereafter the temperature was increased with 10 °C at a rate of 1 °C min^–1^ while measuring the complex modulus G* (γ = 0.01, ω = 1 rad/s). To ensure a stable sample, a temperature ramp was always followed by a time sweep of 30 min at the given temperature (γ = 0.01, ω = 1 rad/s) before performing new frequency and stress relaxation measurements.

#### AFM

AFM images were collected on a Cypher Environmental Scanner from Asylum Research (Oxford Instruments, Santa Barbara, California, U.S.A.) equipped with an NCSTR probe (Nanoworld, Neuchâtel, Switzerland).

### Methods

#### Preparation of Drop Cast Films

All drop cast films were prepared using the following procedure: After synthesis of the polymer, the solution was dried under a N_2_ flow and redissolved in 1,1,1,3,3,3-hexafluoro-2-propanol (HFIP; ABCR) to a concentration of 13 mg/mL. 40 μL of the solution was aspirated and dispensed on a glass round coverslip with a diameter of 14 mm. The solution was left to dry in open air for 1 h before being transferred to a vacuum oven for 24 h at 100 °C to ensure removal of all HFIP.

#### Preparation of Polymer Disks

All disks for rheology were prepared by using the following procedure: After synthesis of the polymer, the solution was dried under a N_2_ flow. The solution was left to dry in open air for 1 h before being transferred to a vacuum oven for 24 h at 100 °C to ensure removal of all solvent. The dried and annealed polymer was cut into small pieces of roughly 0.5 mm × 0.5 mm. 80 mg of these pieces was placed in a iron mold with a disk shape (height = 1 mm, diameter = 8 mm). The mold was placed under a hot press for 3 h at 100 °C and 10 MPa.

#### Cell Culture

Normal human dermal fibroblasts (NHDF; Lonza) were cultured under standard culturing conditions at 37 °C and 5% CO_2_ in Dulbecco’s modified Eagle medium (DMEM) with high glucose, pyruvate, and glutaMAX (Thermo Fisher Scientific), supplemented with 10 v/v% fetal bovine serum (FBS; Greiner Bio-one), and 1 v/v% penicillin/streptomycin (Invitrogen). Cells were harvest every 3 days using trypsin/EDTA (Thermo Fisher Scientific) and cells were used for experiments up to passage 16.

Vitrimer drop cast films were prepared as previously described and placed polymer side up in a 24-well plate and UV sterilized for 20 min. Uncoated glass coverslips were used as a control. To secure the dropcast film, O-rings washed in 70% ethanol were used to surround the coverslip. The well’s plate was then UV sterilized for another 20 min. Cells were harvested using trypsin/EDTA and seeded in a density of 3000 and 10 000 cells per cm^2^ (*n* = 2) in 1 mL of complete medium. Cells were cultured under standard culturing conditions at 37 °C and 5% CO_2_. Cells seeded with 3000 cells/cm^2^ were cultured for 5, 8, and 24 h, whereafter the cells were washed 3× with PBS and fixated at room temperature using 3.7% paraformaldehyde (formalin 37%; Merck) for 10 min. Then 0.5% Triton-X-100 (Merck) in PBS was added to permeabilize the samples. Cells seeded with 10 000 cells/cm^2^ were fixated and permeabilized following the same procedure after 24 h. Then, all samples were washed 3× with PBS and blocked in 10% goat serum in 0.05% Tween (Merck) in PBS for 30 min. Next, the samples were incubated with the primary antibody against vinculin (1:250, anti-vinculin IgG1; V9131, Sigma) diluted in 2% goat serum in 0.05% Tween in PBS overnight at 4 °C. Thereafter, the samples were washed thoroughly with 0.05% Tween in PBS. Next, the samples were incubated with the secondary antibody (1:250, anti-mouse IgG1 (goat), Alexa 647; A21240, Molecular Probes) and phalloidin (1:250, Alexa 488) at room temperature for 2 h. Then, the cells were stained with DAPI in PBS (1:250) for 10 min. Then the samples were washed thoroughly 3× with PBS and mounted cell side down on thin microscope slides using Mowiol. Images were acquired using a Leica TCS SP8 X inverted confocal microscope (Leica Microsystems) using a HC PL APO CS2 objective (20×/0.75). Images were processed in ImageJ and CellProfiler.

## Supplementary Material



## Notes

Experimental details, synthetic procedures, materials and methods, additional spectroscopic and microscopic data, ^1^H and ^13^C NMR results, rheology, AFM and DSC measurements (PDF)
