# Supplementary material for: Engineering Supramolecular Hydrogen Bonding Interactions into Dynamic Covalent Polymers To Obtain Double Dynamic Biomaterials
Source: J Am Chem Soc. 2025 May 23;147(22):18674–84. doi: 10.1021/jacs.4c15102 (PMC12147119; doi:10.1021/jacs.4c15102)
Supplement: Supplementary file 1 [file ja4c15102_si_001.pdf]

SUPPORTING INFORMATION FOR

Engineering supramolecular hydrogen bonding interactions into dynamic covalent polymers to obtain double dynamic biomaterials

Jasper G.M. Aarts[12], Maritza M. Rovers[12], Martin G.T.A. Rutten[12], Patricia Y.W. Dankers*[123]

[1] Institute for Complex Molecular Systems (ICMS), Eindhoven University of Technology, Eindhoven 5600 MB, The Netherlands

[2] Department of Biomedical Engineering, Eindhoven University of Technology, Eindhoven 5600 MB, The Netherlands

[3] Department of Chemical Engineering & Chemistry, Eindhoven University of Technology, Eindhoven 5600 MB, The Netherlands

The compound **UPy-amine** was synthesized previously, according to literature procedure.[1]

S1. Synthesis of 1 and 2

S1.1 Synthesis of 4,4'-(pentane-1,5-diylbis(oxy))bis(2-hydroxybenzaldehyde) (1)

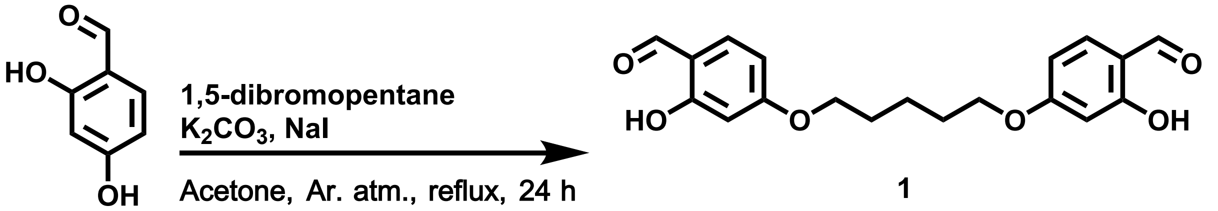

To a thoroughly degassed solution of K_2CO_3 (349 mg, 2.53 mmol) in acetone (2.2 mL) was added 2,4-dihydroxybenzaldehyde (333 mg, 2.41 mmol), 1,5-dibromopentane (264 mg, 1.15 mmol) and NaI (20 mg, 0.13 mmol). The mixture was heated to reflux after which the reaction was allowed to stir for 24 hours under argon. The reaction was cooled to room temperature, the solvent was removed and cold water was added. The organic layer was obtained by addition of ethyl acetate (EtOAc). The aqueous phase was washed 2 times with EtOAc. The organic layers were collected, washed with brine and dried over anhydrous $MgSO_4$. The solid obtained was purified by column chromatography on silica gel with n-heptane/EtOAc as mobile phase (5% to 12%). The pure product was obtained as white solid (324 mg, 0.95 mmol, 82%).

^{1}H NMR (400 MHz, Chloroform-*d*) δ [ppm] = 11.48 (s, 2H), 9.71 (s, 2H), 7.42 (d, *J* = 8.6 Hz, 2H), 6.53 (d, *J* = 8.8 Hz, 2H), 6.42 (s, 2H), 4.05 (t, *J* = 6.3 Hz, 4H), 1.94 – 1.81 (m, 4H), 1.71 – 1.61 (m, 2H).

^{13}C NMR (100 MHz, Chloroform-*d*) δ [ppm] = 195.66, 166.45, 164.36, 135.08, 114.49, 108.94, 101.96, 68.55, 27.77, 22.86.

MALDI-MS: calc exact mass = 344.13 Da, found m/z: 345.12 $[M+H]^+$, 367.11 $[M+Na]^+$, 383.07 $[M+K]^+$.

S1.2 Synthesis of 4,4'-(decane-1,10-diylbis(oxy))bis(2-hydroxybenzaldehyde) (2)

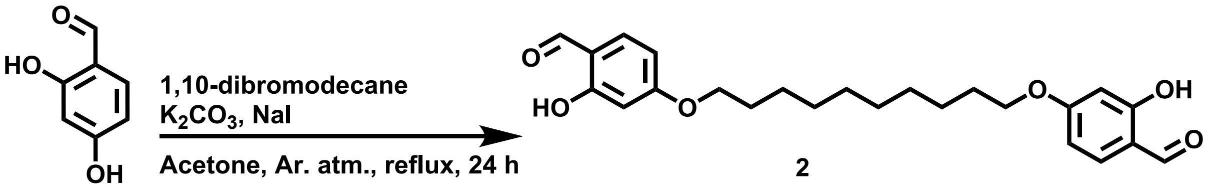

To a thoroughly degassed solution of K_2CO_3 in acetone (7.0 mL) was added 2,4-dihydroxybenzaldehyde (1 g, 7.24 mmol), 1,10-dibromodecane (1.06 g, 3.53 mmol) and NaI (26.5 mg, 0.18 mmol). The mixture was heated to reflux after which the reaction was allowed to stir for 24 hours under argon. The reaction was cooled to room temperature, the solvent was removed, and redissolved in DCM. The solution was washed 3x with water, dried over anhydrous $MgSO_4$, and concentrated. The solid obtained was purified by column chromatography on silica gel with n-heptane/DCM as mobile phase (0% to 50%). The pure product was obtained as white solid (1.26 g, 0.95 mmol, 91%).

^{1}H NMR (400 MHz, Chloroform-*d*) δ [ppm] = 11.48 (s, 2H), 9.70 (s, *J* = 0.6 Hz, 2H), 7.41 (d, *J* = 8.7 Hz, 2H), 6.52 (dd, *J* = 8.7, 2.3 Hz, 2H), 6.41 (d, *J* = 2.4 Hz, 2H), 4.00 (t, *J* = 6.5 Hz, 4H), 1.79 (m, *J* = 14.6, 6.7 Hz, 4H), 1.39 (m, *J* = 46.1 Hz, 12H).

^{13}C NMR (100 MHz, Chloroform-*d*) δ [ppm] = 194.31, 166.45, 164.54, 135.21, 115.03, 108.79, 101.06, 68.58, 29.42, 29.26, 28.91, 25.90.

MALDI-MS: calc exact mass = 414.20 Da, found m/z: 415.20 $[M+H]^+$, 437.18 $[M+Na]^+$, 453.15 $[M+K]^+$.

S2. Synthesis of 2A, 2A-TREN$_{\%}$ and 2A-UPy$_{\%}$

S2.1 Synthesis of linear imine polymers (1A, 2A, 1B, 2B)

Synthesis of the linear imine polymers **1A**, **2A**, **1B**, and **2B** was conducted similar. The general applicable protocol for **2A**: polytetrahydrofuran bis(3-aminopropyl) terminated (10 mg, 0.029 mmol, **A**, M_n = 350 g/mol) and **2** (11.84 mg, 0.029 mmol) were dissolved in $CHCl_3$ (0.4 mL) and the reaction was allowed to stir for 24 hours. The solution was used without further purification.

S2.2 Synthesis of 2A-TREN$_{\%}$

For **2A-TREN$_{20}$**: To a solution of **2A** (15.9 mg) in $CHCl_3$ (0.4 mL), with a reduced amount of **A** (to ensure a final amine to aldehyde ratio of 1:1), was added **TREN** (0.70 mg, 0.0048 mmol) in $CHCl_3$ (0.2 mL). The reaction was allowed to stir for 24 hours and used without further purification.

S2.3 Synthesis of 2A-UPy$_{\%}$

For **2A-UPy$_{20}$**: To a solution of **2A** (20.84 mg) in $CHCl_3$ (0.4 mL), with a reduced amount of **A** (to ensure a final amine to aldehyde ratio of 1:1), was added **UPy-amine** (6.34 mg, 0.0057 mmol) in $CHCl_3$ (0.2 mL). The reaction was allowed to stir for 24 hours and used without further purification.

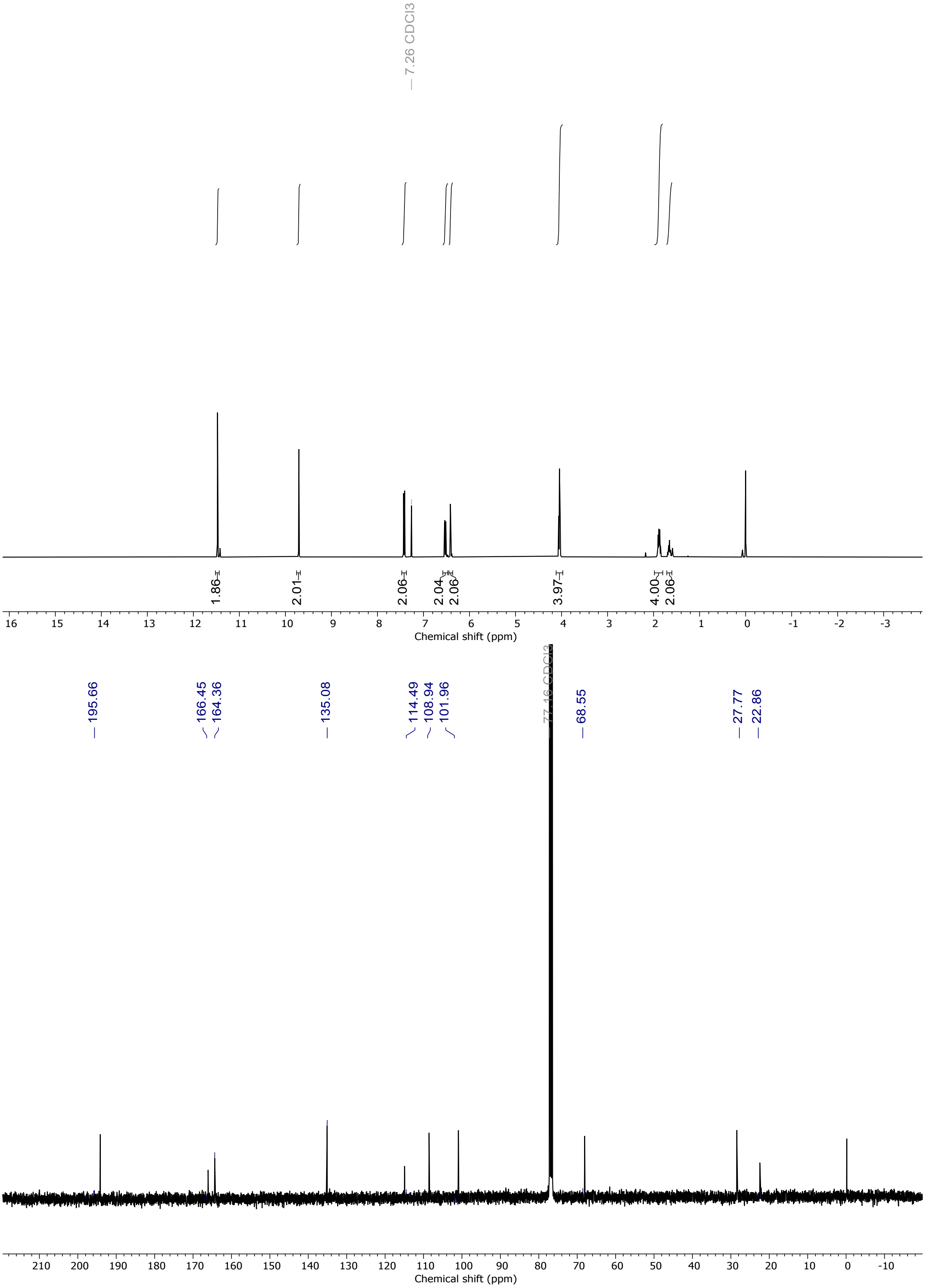

Figure S1. ^{1}H NMR and ^{13}C NMR of monomer **1**.

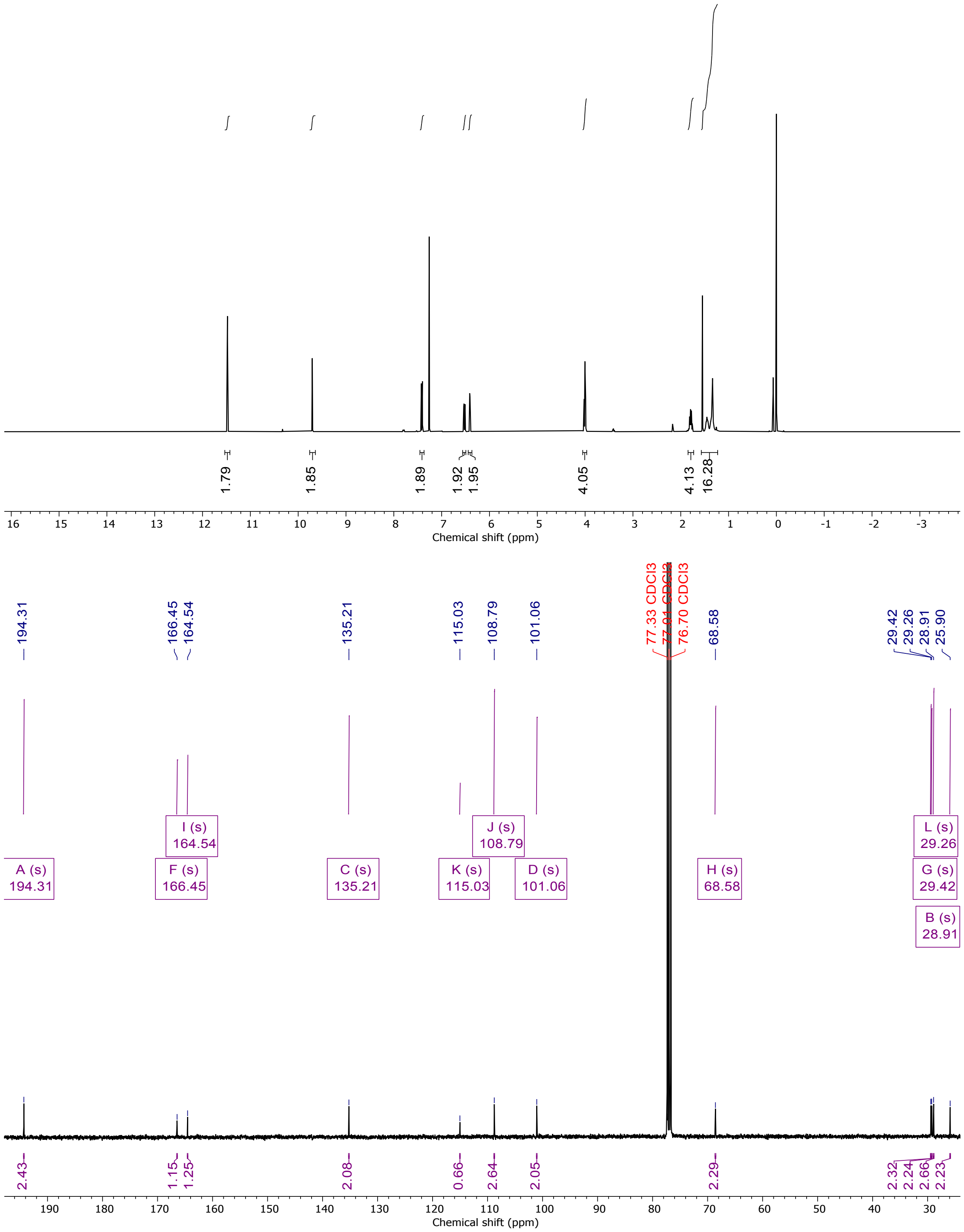

Figure S2. ^{1}H NMR and ^{13}C NMR of monomer **2**.

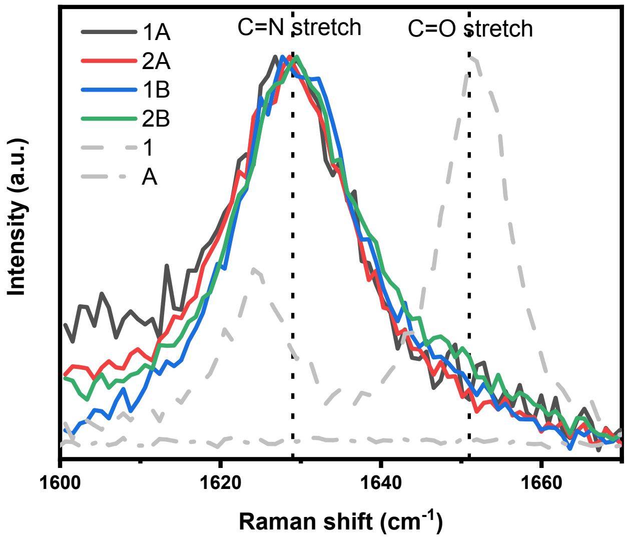

Figure S3. Raman spectroscopy of monomers 1 & A, and linear dynamic covalent polymers **1A**, **2A**, **1B** & **2B**.

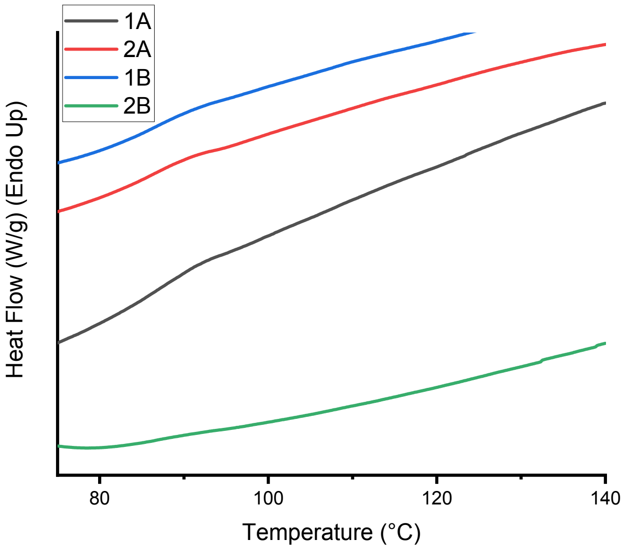

Figure S4. DSC traces (2nd heating run, 40 K/min.) of linear dynamic covalent polymers **1A**, **2A**, **1B** & **2B**.

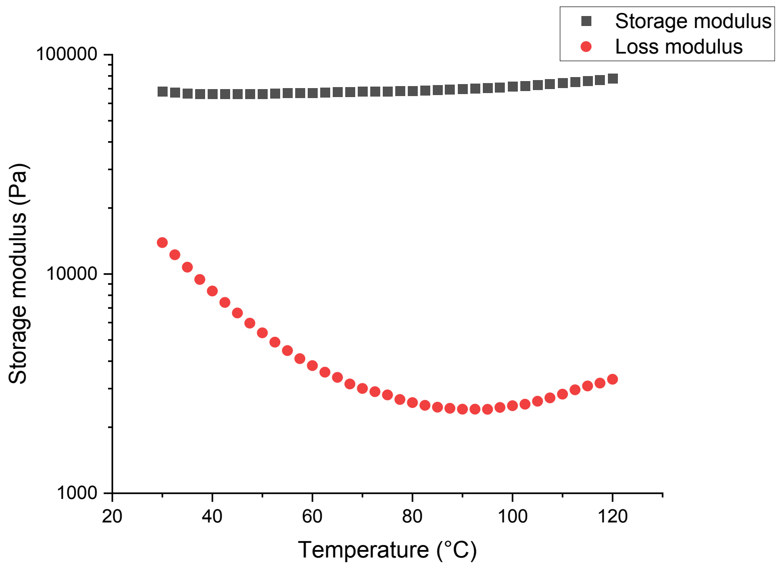

Figure S5. Rheology temperature sweep of **2A**, from 30 °C to 80 °C.

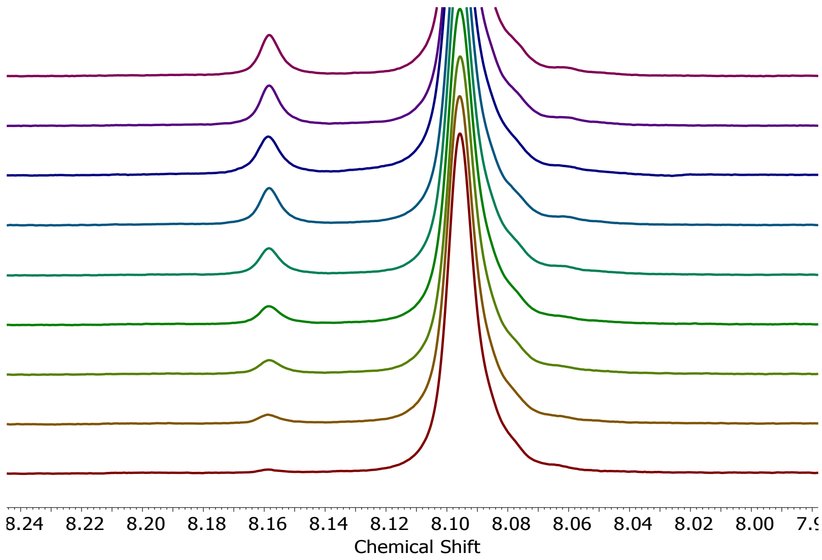

Figure S6. ^{1}H NMR in $CDCl_3$ of 2A-UPy_{20} followed over time (from bottom to top: 5 min. to 24 h)

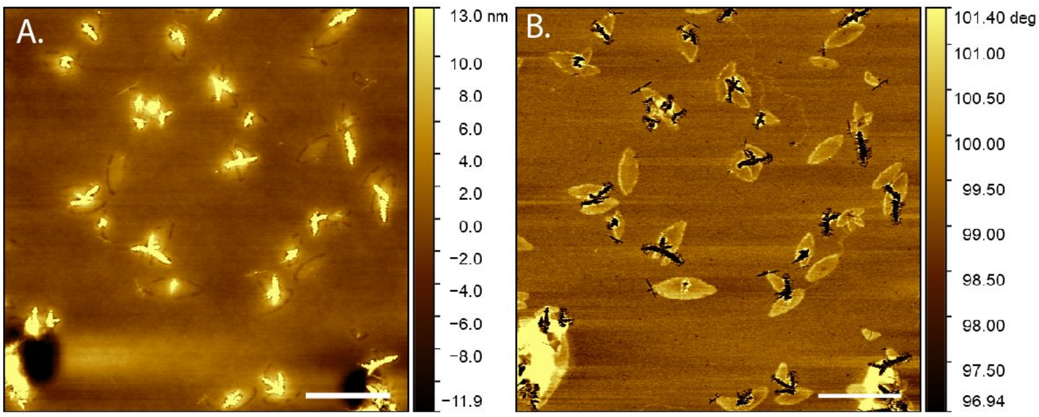

Figure S7. Atomic force microscopy (A) height and (B) phase images of 2A-UPy_{20}.

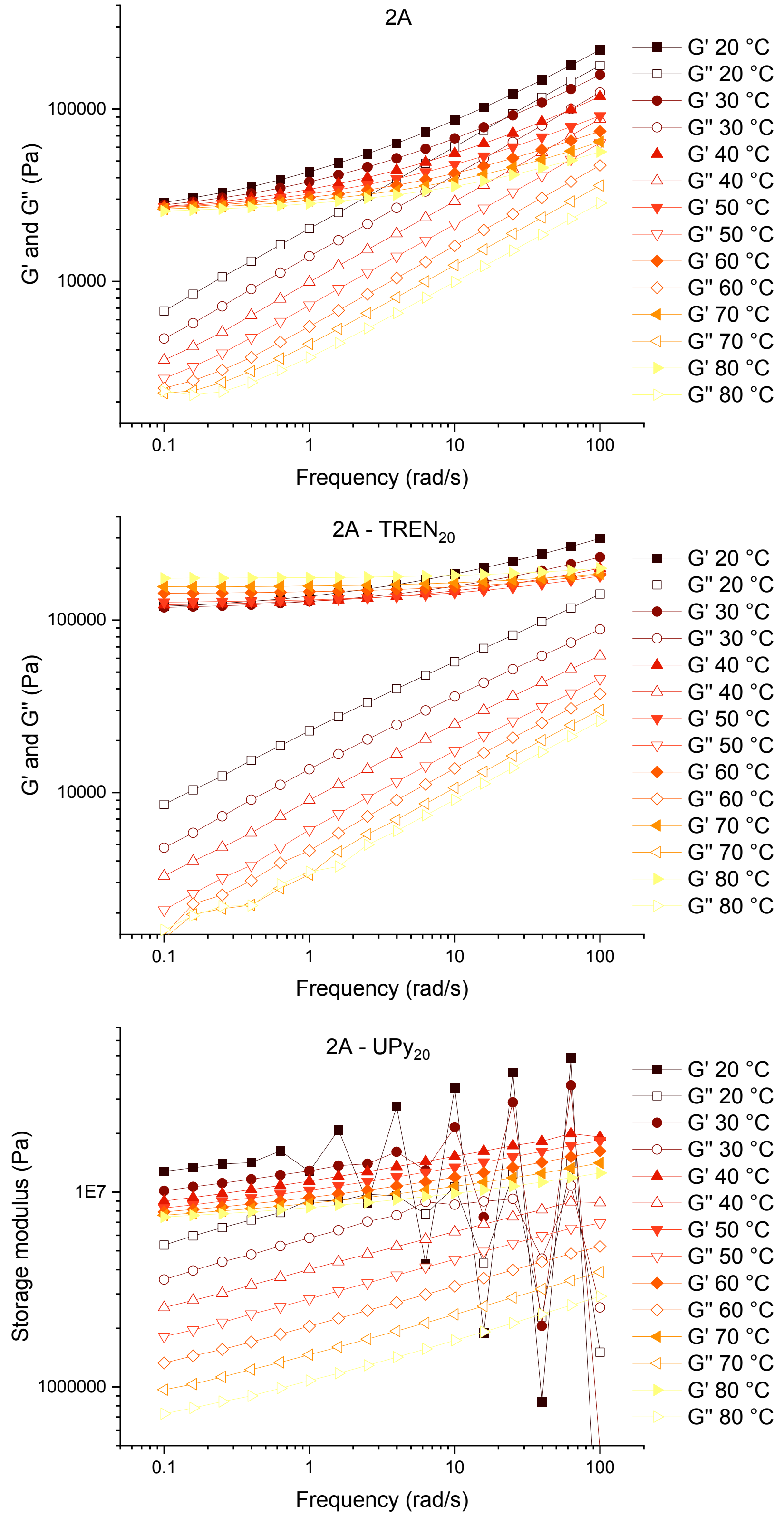

Figure S8. Rheology frequency sweep of **2A** (top), **2A-TREN$_{20}$** (centre), and **2A-UPy$_{20}$** (bottom) at $\omega = 0.1$ rad s^{-1} to 100 rad s^{-1}, at a strain of $\gamma = 0.01$.

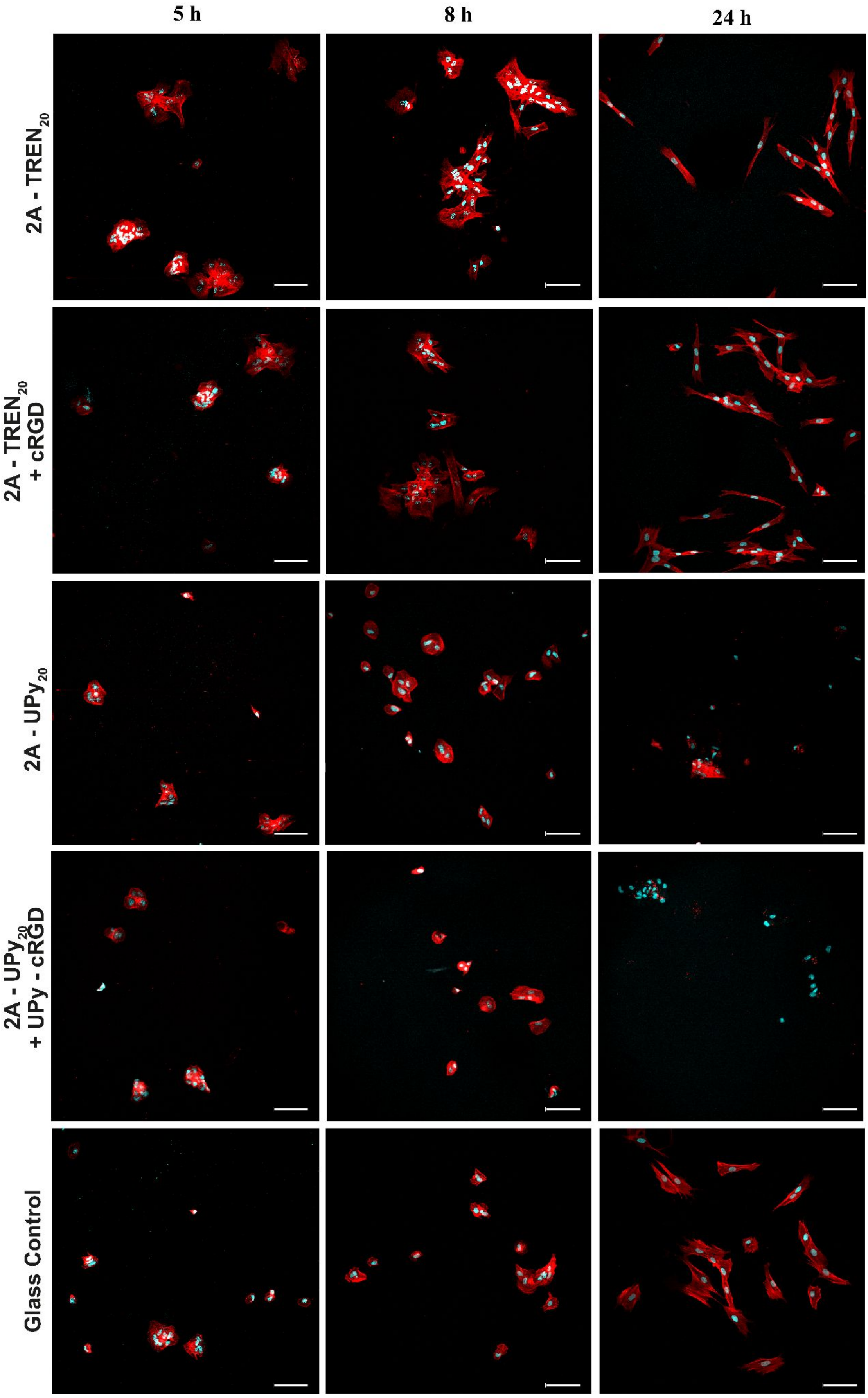

Figure S9. HNDFs cultured for 5, 8, and 24 h on **2A-TREN$_{20}$**, **2A-UPy$_{20}$**, **2A-TREN$_{20}$** + 5 mol % cRGD, **2A-UPy$_{20}$** + 5 mol % UPy-cRGD, and glass with f-actin (red), nucleus (cyan). Scale bar = 100 μm.
